# Parallel Mapping of Antibiotic Resistance Alleles in *Escherichia coli*

**DOI:** 10.1371/journal.pone.0146916

**Published:** 2016-01-15

**Authors:** Sophie J. Weiss, Thomas J. Mansell, Pooneh Mortazavi, Rob Knight, Ryan T. Gill

**Affiliations:** 1 Department of Chemical and Biological Engineering, University of Colorado Boulder, 3415 Colorado Avenue, Boulder, Colorado, 80303, United States of America; 2 Department of Computer Science, University of Colorado Boulder, 1111 Engineering Drive ECOT 717, Boulder, CO 80303, United States of America; 3 Department of Pediatrics, University of California San Diego School of Medicine, 9500 Gilman Drive, MC 0602, La Jolla, CA 92093, United States of America; 4 Department of Computer Science & Engineering, University of California San Diego, 9500 Gilman Drive, MC 0404, La Jolla, CA 92093, United States of America; University of Wisconsin, UNITED STATES

## Abstract

Chemical genomics expands our understanding of microbial tolerance to inhibitory chemicals, but its scope is often limited by the throughput of genome-scale library construction and genotype-phenotype mapping. Here we report a method for rapid, parallel, and deep characterization of the response to antibiotics in *Escherichia coli* using a barcoded genome-scale library, next-generation sequencing, and streamlined bioinformatics software. The method provides quantitative growth data (over 200,000 measurements) and identifies contributing antimicrobial resistance and susceptibility alleles. Using multivariate analysis, we also find that subtle differences in the population responses resonate across multiple levels of functional hierarchy. Finally, we use machine learning to identify a unique allelic and proteomic fingerprint for each antibiotic. The method can be broadly applied to tolerance for any chemical from toxic metabolites to next-generation biofuels and antibiotics.

## Introduction

Chemical genomics, or the study of the genome-scale response to small molecules, has rapidly advanced thanks to synthetic biology approaches. For example, studies of phenotype mapping of small molecule landscapes have led to elucidation of novel genetic functions and drug mechanisms [1–3]. These pioneering studies took large genomic libraries, usually painstakingly created [4, 5], and characterized them under a range of chemical and physical conditions using DNA microarrays. Studies of chemical tolerance have also used adaptive evolution methods to identify mutations that contribute to fitness [6, 7]. While these studies closely mimic responses to stresses in nature, the extent of genotyping is limited by the throughput of whole-genome sequencing.

The increasing throughput and decreasing cost of multiplex oligonucleotide synthesis [8] and high-throughput sequencing [9] has enabled unprecedented advances in throughput of genome engineering and analysis technologies [10–13]. For example, recent studies have leveraged high-throughput sequencing to expand the characterization of yeast deletion libraries [14]. Along these lines, we recently reported the trackable multiplex recombineering (TRMR) approach [15]: a one-pot construction of a barcoded, genome-scale library simulating overexpression and knockdown of over 4,000 genes in the Gram-negative bacterium *E*. *coli*. Initial experiments with the library focused on the genomic response to various carbon sources and biofuel-related inhibitory conditions using DNA microarrays and exploratory by-hand analyses [15, 16].

At sub-lethal antibiotic concentrations such as those found in wastewater and agricultural runoff, the contribution to microbial fitness of cellular factors is not nearly as well-studied [17] as horizontal gene transfer of specific resistance effectors [18]. Thus, understanding the response and resistance of microbes to antimicrobial compounds is of critical importance. To isolate gene products contributing to antibiotic resistance, several genomic and proteomic studies have been performed [19–23]. However, previous attempts to characterize genome-scale responses to antibiotic challenges [1, 3, 6, 7, 24–26] relied on either (1) the low-throughput construction of large libraries or (2) many generations of adaptive evolution, where characterization was limited by sequencing surviving colonies.

Here we report a method for the rapid and deep characterization of laboratory population dynamics in response to eight antibiotics by multiplex selection, next-generation sequencing, and multivariate analysis of *E*. *coli* TRMR libraries. Our findings support the development of multi-drug resistance and susceptibility genes as an important step in the evolution of antibiotic resistance in microbial populations at sub-lethal concentrations. Finally, to expand the throughput and extent of our bioinformatic analysis, we integrate the data gathered into the QIIME multivariate analysis pipeline, with which we examine the response at a pathway level and identify a unique genomic signature for each antibiotic.

## Methods

### Strains and plasmids

The TRMR library was previously constructed [15]. Briefly, *E*. *coli* MG1655 (ATCC #700926) cells were subjected to multiplex recombineering using synthetic DNA cassettes containing either an “up” (strong promoter and RBS) or “down” (no promoter or RBS) phenotype with homology regions corresponding to over 4,000 genes in the *E*. *coli* genome. The synthetic cassettes also contained unique barcodes for rapid characterization and gene-trait mapping. In this study, a modified version of strain JWKAN, which is MG1655 with the kanamycin resistance gene *neoR* (from pKD13 [27]) inserted in a safe region and barcoded as in the rest of the library, was used as the wild-type control. Expression of FLP recombinase (pCP20 [28]) excised *neoR* from the genome using flanking FRT sites to create a barcoded MG1655 without kanamycin resistance, which we refer to as MG1655-BC. This phenotype was confirmed by replica plating and the genotype confirmed by colony PCR.

### Antibiotic MIC Determination

Overnight cultures of MG1655-BC cells were subcultured into various concentrations of antibiotics in MOPS media [29] at 37C to determine the minimum inhibitory concentration (MIC) for each compound. All antibiotics were purchased from Sigma-Aldrich (St. Louis, MO). The MIC for each antibiotic was determined by an iterative process using the procedures and definitions of Andrews [30]. First, an estimate was determined by growing MG1655-BC in 96-well plates in triplicate in 2-fold increments around the MIC found in the literature (if any) [30]. The 2-fold determined MIC was then refined by growth in 1.2 fold increments. The refined MIC was used for liquid culture in MOPS media in 250 mL flasks, inoculated at OD_600_ 0.02 with MG1655-BC or the recovered TRMR library. The final MIC concentration was determined to be the concentration at which MG1655-BC showed no growth and the TRMR library showed significant (OD_600_ > 0.2) growth at 24 hours.

### Cell culture and selection conditions

The TRMR “up” and “down” libraries were recovered from frozen stocks by inoculating glycerol stocks of the constructed libraries in low salt LB media with 90μg/mL blasticidin-S to OD_600_ 0.4. The cells were grown at 37C in a shaking incubator to an OD_600_ of approximately 0.8.

When the initial TRMR and MG1655 cultures reached the desired OD_600_, they were transferred to two identical sets of three selection flasks containing 50 mL MOPS media at 80% of the previously determined MIC (sub-inhibitory selection concentration, SSC) for each of the eight antibiotics (for 48 flasks total) tested to an OD_600_ of approximately 0.02 [15]. TRMR “up” and TRMR “down” libraries were added in equal amounts as determined by OD. These initial cultures were then harvested by centrifugation and frozen as pellets for initial concentration values, which we refer to as time point zero. Growth proceeded under antibiotic selection conditions at 37°C and cells were harvested by centrifugation after 24 hours and upon reaching a 1.5 OD_600_.

### Antibiotic Colony Sequencing

Individual colonies from each selection were amplified including the barcode tags by PCR. All PCRs were performed using Phusion polymerase (NEB). The PCR product was confirmed to correspond to the barcode region by gel electrophoresis. The DNA was then purified using a QIAquick gel extraction kit (Qiagen), and sent for Sanger sequencing (MWG Eurofins Operon). The incorporated tag sequence was compared with S1 Table of Warner et al. [15] to identify alleles. For high-throughput sequencing, the genomic DNA from 10^9^ cells from all the selections was extracted using the DNeasy Blood & Tissue Kit (Qiagen). Four base-pair tags were appended using PCR near the beginning of each TRMR-unique barcode to further distinguish the samples by replicate. PCR products of roughly 180 bp were gel-extracted and purified using the QIAquick gel extraction kit (Qiagen), and combined in equimolar amounts. The resulting mixture of amplicons from all replicates and time points for each antibiotic sample was assigned a unique Illumina index and prepared for sequencing according to Illumina TruSeq 1x50 guidelines [31] and sequenced on an Illumina HiSeq 2000.

### Sequencing Data Analysis

Each FASTQ file produced by the high-throughput sequencing was read and signal quality filtered in parallel using a custom MATLAB script. The 50 base pair reads were matched to 50 base pair DNA sequences in a mapping file corresponding to the expected barcodes in genomic context. These sequences included a four base pair tag for replicate and experiment identification, as well as the unique TRMR tag sequences for each gene as found in S1 Table of Warner et al [15]. Any FASTQ sequence not matching those in the mapping file within 1 base pair was discarded to allow distinguishing between the replicates while minimizing spurious mapping of sequences to genes. This strict quality filtering meant 10–40% of the sequences in each FASTQ file were retained. The discarded sequences represent sequences with more errors, e.g., miscalls. The raw number of counts for each sample, presented in table format, can be found in S1 Dataset.

Inherent bias in construction and finite reads meant that not every allele appeared in the naïve (unselected) cases. Thus enrichment in this study was defined as the relative increase in a particular allele after selection with respect to the population before antibiotic was applied (time point zero [TP0]) according to the following formula for enrichment of a given allele A.

$$enrichment_{A}={\left(\frac{counts_{A}}{\sum_{n}counts_{n}}\right)}_{selection}-{\left(\frac{counts_{A}}{\sum_{n}counts_{n}}\right)}_{TP0}$$

The “top” alleles described are the alleles in each selection case with the highest enrichment over the naïve case.

### Bioinformatic Analysis

Most analyses were performed using the QIIME (Quantitative Insights into Microbial Ecology) pipeline, version 1.7.0 [32]. The open-source QIIME pipeline was built using the PyCogent libraries [33] and the Python programming language. Once installed, QIIME analyses are performed though a simple command-line interface, where the input and output file paths are specified, as well as any method parameters. QIIME was used for all of the following analyses: normalization, formation of a distance matrix, principal coordinates analysis (PCoA), Procrustes analysis, supervised learning, part of the network analysis, COG relative abundance plots, and ANOSIM. The QIIME scripts used for the above and below list of analyses were: single_rarefaction.py, normalize_table.py, beta_diversity.py, principal_coordinates.py, transform_coordinate_matrices.py, supervised_learning.py, make_otu_network.py, summarize_taxa_through_plots.py, and compare_categories.py. All of these QIIME scripts use as input the table of gene counts in each sample, and corresponding metadata, found in S1 Dataset, or products from previously used scripts (e.g. beta_diversity.py should be used before principal_coordinates.py).

First, the tables in S1 Dataset were normalized. Normalization is necessary to correct for uneven library sizes, as well as other artifacts of the sequencing process [34]. The tables were subsampled (rarefied) to a depth of 2000 sequences per sample. Another normalization method implemented in R and QIIME, metagenomeSeq’s cumulative sum scaling (CSS), was performed in order to ensure robustness of results [35]. Next, a distance matrix was formed using Bray-Curtis dissimilarity [36, 37], since antibiotics selecting for the same genes should be deemed more similar, and because Bray-Curtis is less sensitive to data sparsity and compositionality [38–40]. Then, PCoA was performed on the distance matrices. We also assessed the results using Euclidean and binary Jaccard metrics with similar results.

Procrustes analysis, which enables comparison of the relative distances between points in two multivariate datasets, [41] was also performed on the gene and COG distance matrices. The measure of fit (M^2^) was calculated as the sum of the squared distances between corresponding sample points after the data is translated, rotated, and scaled to minimize the distance between the two datasets. The p-value was calculated by 1000 Monte-Carlo permutations in which the sample labels were randomly permuted; the number of iterations in which the M^2^ value was lower than the actual was divided by 1000 to yield the p-value.

Supervised learning was performed in QIIME on the two tables in S1 Dataset using the random forest machine learning method [42], with 5,000 sequences per sample, 500 trees, and leave-one-out cross-validation to estimate the generalization error and feature importance [43, 44]. Plots of alleles based on genomic location were generated using Circos software [45]. Genes were annotated with their corresponding Clusters of Orthologous Groups (COGs) [46]. Relativized counts were plotted using the summarize taxa scripts in QIIME [32]. Networks were constructed using Cytoscape [47]. ANOSIM was also carried out in QIIME [48] using the ‘vegan’ package in R [49].

## Results and Discussion

### Selection of antibiotic-resistant alleles from a genome-scale library

**Fig 1:**
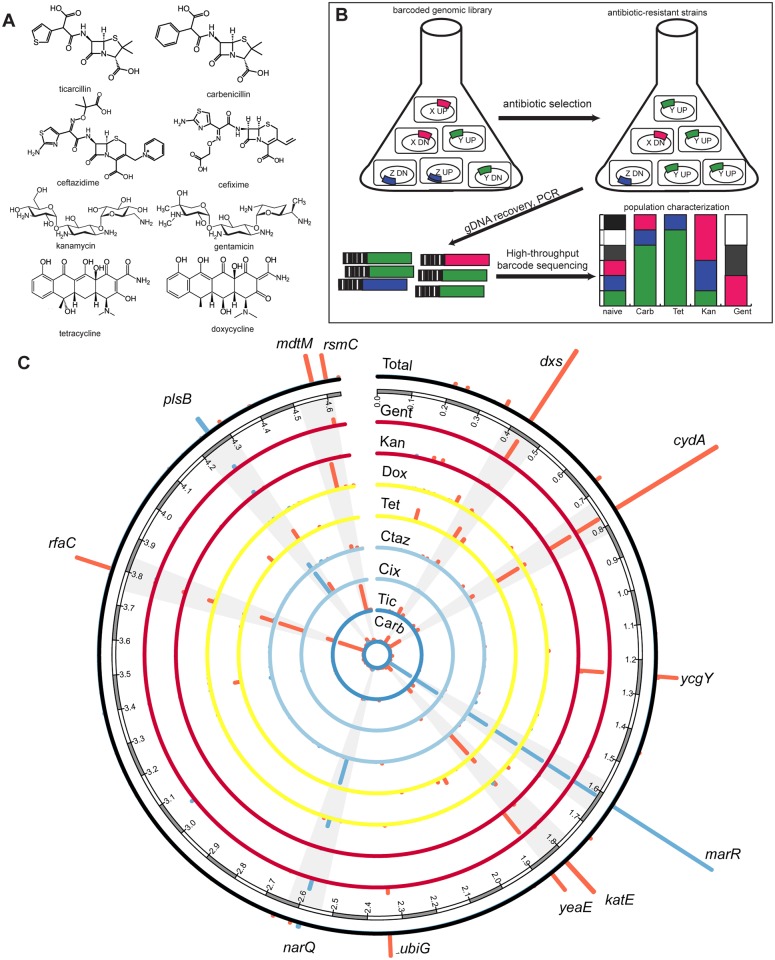
Selection of a genome-scale library on several antibiotics yields multi-drug resistant genes. (a) Chemical structures of the eight antibiotics used in this study. (b) The TRMR library containing strains simulating “up” or “down” expression phenotypes in *E*. *coli* is grown in selective conditions. The genomic DNA of the survivors is harvested and amplified by PCR and the amplicon is sent to high-throughput sequencing, after which it is analyzed. (c) Enrichment scores for TRMR “up” (blue) or “down” (red) alleles for particular antibiotics are plotted relative to their location in the *E*. *coli* genome (in Mb). Alleles enriched in many or all selections are highlighted. The outside ring represents a linear combination of all eight antibiotic trials.

We subjected our genome-scale, barcoded library to selection on eight different antibiotics with three different mechanisms of action (S1 Table). Pairs of antibiotics were selected for chemical similarity (e.g., ticarcillin differs from carbenicillin only by the substitution of a five-membered thiophenyl moiety for a benzyl moiety) (Fig 1a). Briefly, *E*. *coli* MG1655 cells were previously subjected to multiplex recombineering using synthetic DNA cassettes containing either an “up” (strong promoter and ribosome binding sequence [RBS]) or “down” (no promoter or RBS) sequence along with homology regions corresponding to 4,077 genes in the *E*. *coli* genome. The synthetic cassettes also contained unique barcodes for rapid quantification of each of the approximately 8,000 TRMR mutants by microarray or pyrosequencing technologies (S1 Fig).

To design our growth selections, we first measured the minimum inhibitory concentration (MIC) for each antibiotic of interest in a strain equivalent to the parent strain of the TRMR library. MG1655-BC, a version of MG1655 with a barcode inserted at a silent site (the *attn7* site), was grown in liquid culture in triplicate at varying amounts of antibiotic to determine the concentration at which growth of the wild-type strain was inhibited (S1 Table). We were initially surprised by the MIC of MG1655-BC cells with respect to the β-lactam antibiotics (e.g., 44 μg/mL for carbenicillin, given a typical working concentration of 50 to 100 μg/mL for *E*. *coli*). However, further investigation of the literature revealed that the MIC of *E*. *coli* for carbenicillin is highly strain-dependent. According to the Antimicrobial Index [50], MICs of carbenicllin for strains of plasmid-free *E*. *coli* can measure from 1.56 to 32 μg/mL. In addition, a recent report demonstrated that β-lactam resistance in MG1655 is particularly susceptible to sugar levels and inoculum [51], which was consistent throughout all experiments in this work. The MICs of all other antibiotics were within ranges that have been reported in literature for MG1655 [50].

Once the MIC was determined, the TRMR library was inoculated in triplicate in two identical sets of flasks containing MOPS rich defined media and one of eight antibiotics of interest at 80% of the MIC (48 flasks total). We performed selections at these concentrations in an attempt to normalize the selective pressure across all antibiotics. These flasks were grown until the late exponential phase with samples extracted at 24 hours and upon reaching late exponential phase. Genomic DNA was extracted and used as a template for PCR amplification and preparation for Illumina HiSeq sequencing of the barcode region (Fig 1b). More than 22 million barcode reads were counted and assigned to individual clones and degree of enrichment (see Methods) were calculated for all 8,077 TRMR mutants in each of the selections performed (Fig 1c). This analysis identified alleles enriched in TRMR libraries after selection that are consistent with previous studies on antibiotic resistance, plus uncharacterized genes potentially involved in resistance that could be important for further study (S2 Table). In addition, we use enrichment measurements to report alleles that may confer hypersensitivity (S3 Table, S2 Fig).

### Alleles contributing to antibiotic resistance

Our data suggest that multi-drug resistance alleles are consistently enriched regardless of the antibiotic selection performed (Fig 1c), and comprise a large fraction (10–90%) of each of the selected populations (S3 Fig). Specifically, we found five alleles that were enriched in all eight selections and six that were enriched in all but one case. These 11 alleles comprised over 30% of the selected population in six cases, but comprised only 0.6% of the population before selection. These results suggest that laboratory selections enrich for MDR alleles (generalists), and not only for distinct sets of individual antibiotic resistance alleles (specialists). It is important to note that previous selections of the TRMR library on the same media without antibiotics [15] did not result in significant enrichment of any of the below noted alleles (i.e., all rank below the 100 most highly enriched in MOPS media alone).

One of the most prevalent alleles, occurring in the ten most highly enriched alleles in all cases (Fig 1c) is *marR*_up. In this construct, the *marRAB* (where *mar* stands for “multiple antibiotic resistance”), which is normally negatively autoregulated by *marR* [52], is under control of the TRMR strong and constitutive promoter (pL_tetO_). *MarA* is known to regulate several genes involved in resistance to antibiotics and multidrug efflux [53]. The *rfaC_down* strain occurs in the ten most highly enriched alleles in seven of the eight cases. In this mutant (and all other TRMR “down” mutants), the native RBS has been removed to minimize translation. *RfaC* catalyzes a key step in lipopolysaccharide synthesis [54]. *RfaC* mutants in several pathogenic bacteria including *E*. *coli* show increased resistance to various antibiotics [55]. It is not clear why the “down” mutation was selected (as opposed to the “up” mutation). However, because the blasticidin resistance cassette contains a strong EM7 promoter 5’ of the gene of interest (S1 Fig), it is possible that some read-through may occur, leading to constitutive downstream expression.

Other alleles consistently enriched by selection with several antibiotics and previously associated with antibiotic resistance included genes related to (1) managing oxidative stress: *katE* [56] and *sodC* [57], (2) oxidative phosphorylation: *cydA* [10], (3) transport and efflux: *mdtM* [58], and (4) other metabolic processes: *dxs* [59], and *plsB* [60]. We then confirmed that apparent increased antibiotic resistance led to increased growth on many antibiotics. S4 Fig shows the 24 hour OD_600_ on various concentrations of all antibiotics for several isolated individual strains with their enrichment scores for comparison (S4 Fig). In addition to genes enriched in multiple selections, we identified a range of genes enriched in individual selections and several genes of unknown or uncharacterized function (S2 Table). The Additional Data file lists allele counts for all selections in this work.

Among the genes enriched in individual selections was *rsmC*, a 16S ribosomal subunit nucleotide methylase. The *rsmC_down* allele was highly enriched in the gentamicin selection. Interestingly, a recent study implicates 16S ribosomal RNA methylases in aminoglycoside resistance in *Enterobacteriaceae* [61]. A highly enriched allele for cefixime resistance was *mreC_up*. *MreC* is a rod-shape determining protein involved in peptidoglycan synthesis that has been associated with β-lactam resistance in *Helicobacter pylori* [62]. *SecD*, another allele isolated in the cefixime selection, has also been linked to β-lactam resistance in *E*. *coli* [55].

Unexpectedly, several enriched alleles for cefixime, ticarcillin, and gentamicin selection(s) corresponded to hydrogen production and formate processing including *fdnG*, *hyfJ*, and *narQ*. It is possible that the actions of these proteins affect the proton motive force, either facilitating increased drug efflux by increased PMF or decreasing drug uptake by reducing PMF (as is well known to affect the toxicity of charged compounds such as aminoglycoside antibiotics [63]). Several alleles were isolated that correspond to genes with unknown functions. They include: *ycjO* (putative ABC transporter), *yiiR*, *ybeT* (conserved outer membrane protein), *yafL* (inner membrane protein), *ycgY*, *yeaE* (methylglyoxal reductase), *yebY*, *yigB*, *yiiR*, and *yncH*. The contribution of these genes to antibiotic resistance warrants further investigation.

Finally, the targeting of antibiotic sensitivity genes provides a possible mechanism to treat resistant infections. To determine genes that might convey sensitivity to the antibiotics of interest, we also recorded the alleles with the lowest degree of enrichment (i.e., largest decrease in frequency throughout a selection) (S3 Table). Our analysis suggested considerable overlap in susceptibility genes across the antibiotics investigated (S2 Fig). Many of the proteins encoded by these alleles are targeted to the inner membrane. Previous experiments also showed that these specific alleles grew well on non-selective rich MOPS media [15]. Thus, it is possible that changes in expression of these inner membrane proteins alter the overall inner membrane fluidity or porosity, allowing antibiotics to traverse membrane more easily. While this possibility should not be discounted, it should also be noted that all of the above susceptible alleles were present in large quantities at time point zero. Given the strength of each selection, it is possible that these alleles were simply diluted down to the limit of detection. This is an issue of selection design; in particular our designs were targeted at enrichment for resistance phenotypes as opposed to identification of susceptibility phenotypes.

### Allelic responses to chemically similar antibiotics are weakly dissimilar

**Fig 2:**
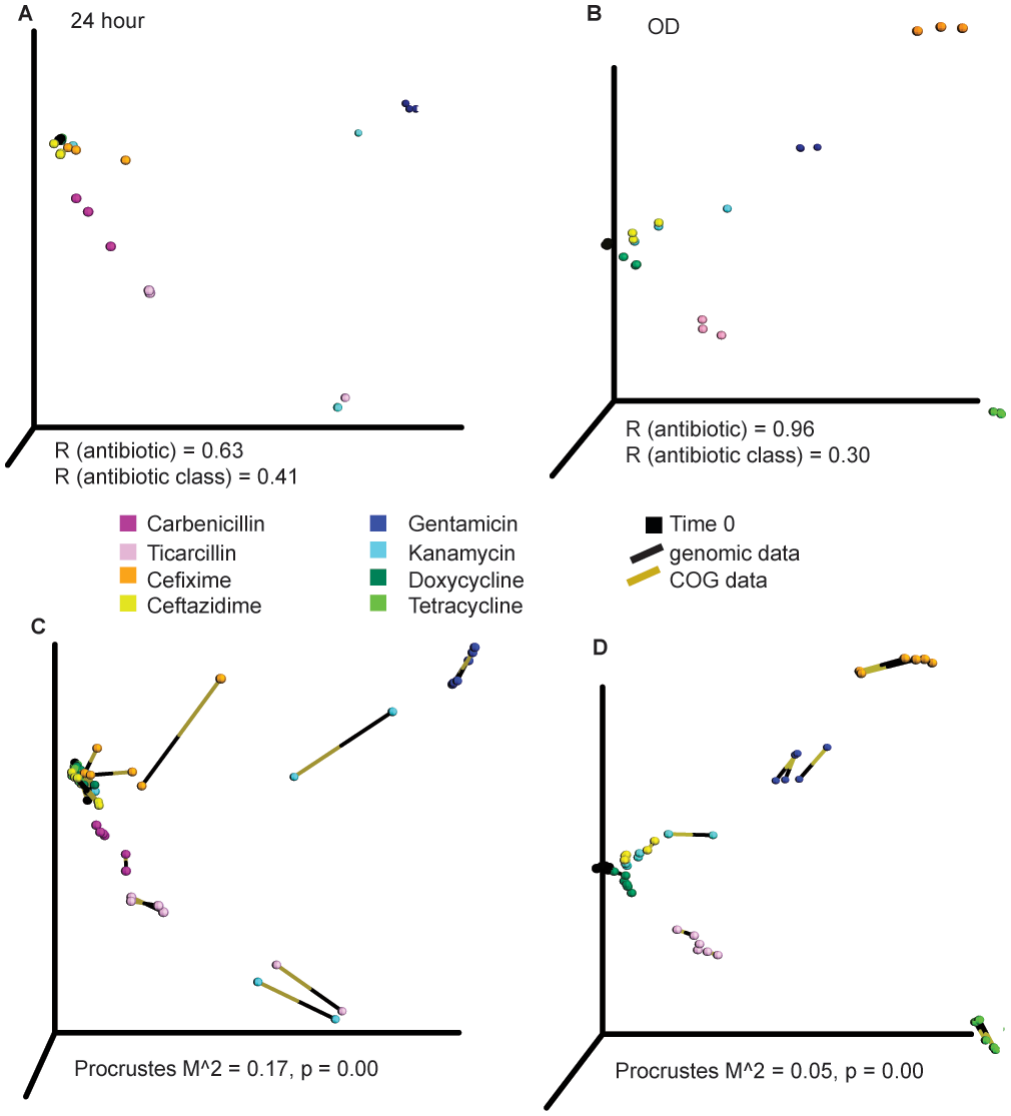
Separation of antibiotic classes in PCoA space is weak across multiple levels of functional hierarchy. (a,b) PCoA analysis, using Bray-Curtis distance, of the antibiotics at (a) 24 hours (b) upon reaching the late exponential phase (OD). ANOSIM R-values are plotted for separation by antibiotic or by mechanism of action. (c,d) Procrustes analysis indicates significant alignment between the COG (gold end of the line) and gene (black end of the line) PCoA profiles in the 24 h and OD selections. The longer the line connecting the COG and gene points, the less aligned the two points are in PCoA space, increasing the stress value (M^2^).

Our data suggested that sub-lethal antibiotic treatment strategies selected for multi-drug resistance alleles. To explore this suggestion in more depth, we performed principal coordinate analysis (PCoA) on all replicates from each selection. PCoA allows for visualization of multi-dimensional variables in 3D space by condensing distance metrics into the most important coordinates while minimizing the loss of information. We specifically hypothesized that antibiotics with similar chemical structure and belonging to the same class (e.g., gentamicin and kanamycin) would present a similar allelic response and therefore cluster together in PCoA space, and that antibiotics having similar mechanisms of action (e.g., the aminoglycosides and the tetracyclines, which both act by binding 30S ribosomal subunits) would as well. Although some patterns appear at 24 hours and after reaching the late exponential phase (Fig 2a and 2b), such as the location of gentamicin and kanamycin in the upper half of PCoA space, other patterns are unexplained. For example, doxycycline, carbenicillin, and ceftazidime cluster near time point zero. This finding is supported by a weaker ANOSIM R value for antibiotic class or mechanism of action (Fig 2a and 2b). ANOSIM R-values near zero indicate random grouping. Network analysis, in which samples sharing similar genes are drawn together, confirms that the subtle antibiotic PCoA clustering patterns, as there are no large differences between antibiotic types (S5 Fig). However, differences between antibiotics are discernable by ANOSIM [64], which is an extremely sensitive test (S4 Table). These results are robust to normalization technique, replicate, and distance metric (S6 and S7 Figs).

### Clusters of Orthologous Groups analysis elucidates functional hierarchy

**Fig 3:**
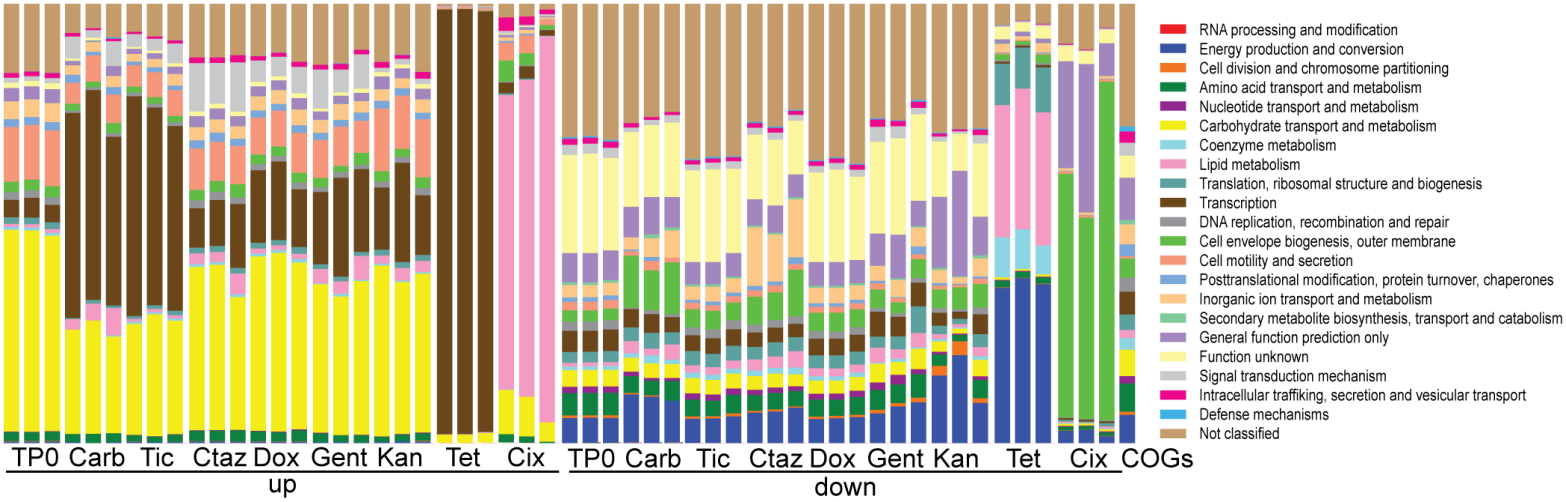
Clusters of Orthologous Groups (COGs) relative abundance of selected populations. Label format is Antibiotic_replicate_up/down, e.g., CarbOD_1_up means COG counts for carbenicillin, replicate 1 of 3, and TRMR “up” alleles. Antibiotic abbreviations: Carbenicillin (Carb), Ticarcillin (Tic), Ceftazidime (Ctaz), Cefixime (Cix), Gentamicin (Gent), Kanamycin (Kan), Doxycycline (Dox), and Tetracycline (Tet). Far right bar: COG distribution as represented in the wild-type *E*. *coli* genome.

Although antibiotics of similar classes or targeting the same complex did not exhibit significant clustering in PCoA space at the specific allele level, we speculated that clearer patterns might be revealed when the PCoA analysis was performed at the level of encoded functions. To gain an understanding of mechanisms of action on a pathway level [65], a matrix of clusters of orthologous groups (COG) [46] was formed by summing the counts of genes belonging to the same COG in the same sample. We then performed Procrustes analysis to analyze the similarity of the gene and COG distributions in PCoA space (Fig 2c and 2d). Procrustes analysis stretches, rotates, and scales two datasets to determine if similar conclusions could be drawn [66]. The p-values are less than 0.001, suggesting that the functional profiles could be predicted from the TRMR alleles enriched by selection because both matrices display similar PCoA clustering patterns. This match between COG and gene distributions implies that selection acts in broadly similar ways at multiple levels of the functional hierarchy. Fig 3 shows the similar COG distribution of the antibiotic samples over time. However, the “up” and “down” allelesperform very different functions, and are different from the wild-type distribution (far right bar). The TRMR “up” alleles were enriched in COG categories of carbohydrate metabolism and transport (yellow), and transcription (brown). The latter was expected for the tetracyclines and aminoglycosides as ribosomal inhibitors, but not for β-lactams. The functions of the TRMR “down” alleles were much more diverse.

### Supervised learning distinguishes resistance “fingerprints”

**Fig 4:**
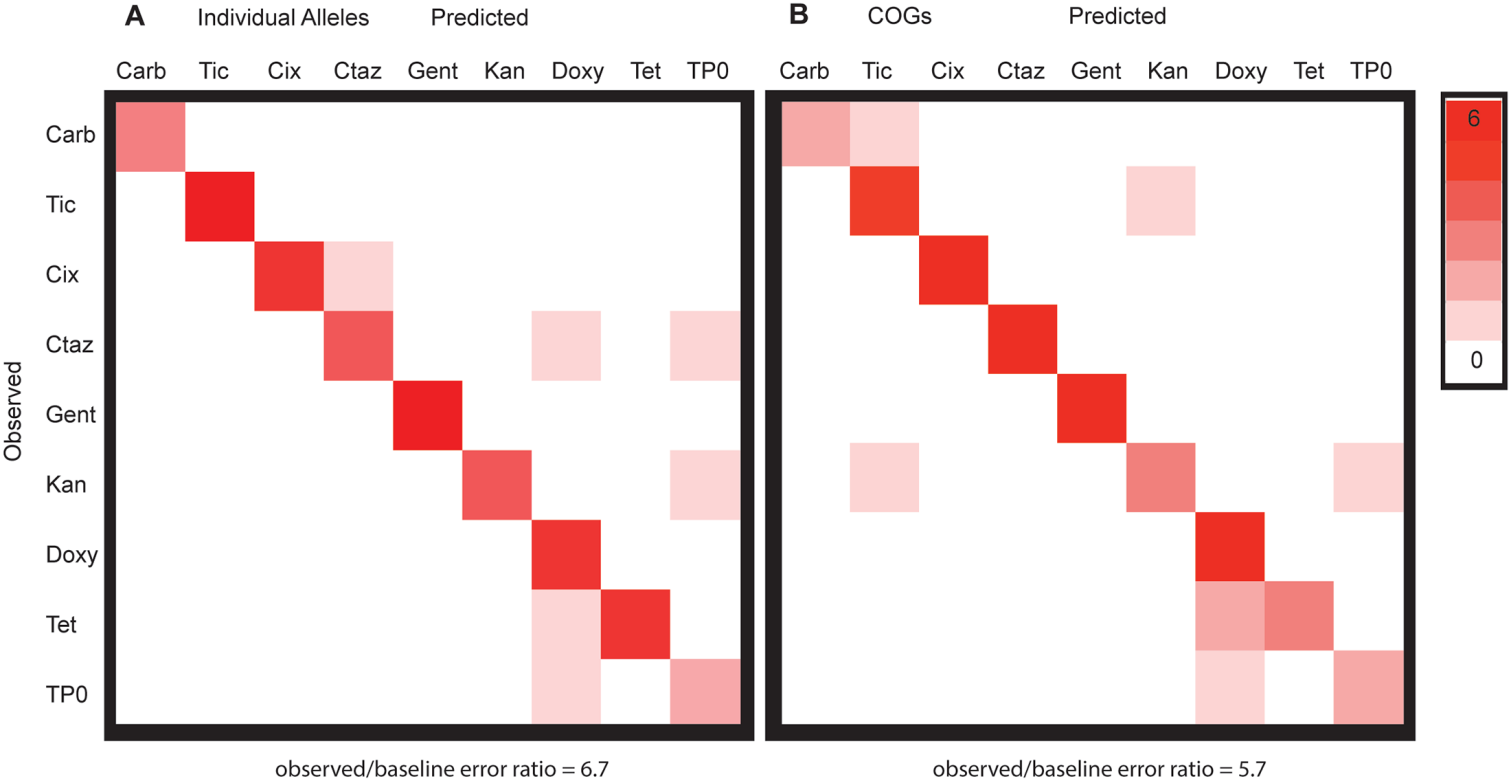
Supervised learning is able to distinguish between the antibiotics at both the allele and COG level. Confusion matrices for random forest classifiers. Off diagonal classification represents classifier error. Antibiotic abbreviations as in Fig 3.

Given that MDR alleles were a significant fraction of every selection (S3 Fig), we wanted to understand whether the final antibiotic populations could be distinguished. To do so, we used supervised learning to identify combinations of genes that may be unique to individual antibiotics, and thus represent a genomic “resistance fingerprint” for each antibiotic. We used the random forest classifier [42] to generate confusion matrices from 48 samples (24 hour and late-exponential phase selections, in triplicate, on each of eight antibiotics), which indicate true vs. predicted classifications when a portion of the dataset is withheld from model training (Fig 4). At the level of individual alleles, it was difficult to distinguish between some antibiotics (as shown by shading off of the diagonal), especially between antibiotics of the same class or mechanism of action (Fig 4a). The random forest classifier returns a ratio of baseline error to observed error of 2.2, indicating that the classifications are estimated to be 2.2 times more accurate than random guessing, a statistically significant but weak effect.

However, when alleles are grouped by COG category, supervised learning improves substantially. There is excellent classification of antibiotics with a baseline error ratio of 5.7 (Fig 4b). This indicates that each antibiotic has a unique signature at the COG level. Classification between the antibiotics may further improve by adding more antibiotics within each class. This hypothesis is supported by perfect distinction (baseline error ratio 24.0) between gentamicin and kanamycin, antibiotics of the same class with similar chemical structure, in a separate detailed time course selection (S8 Fig). Furthermore, the subtle genetic differences arise at the first antibiotic application, independent of selection length (S8 Fig).

The genes, COGs, and the enrichment patterns the random forest classifier uses most to distinguish between the antibiotics are found in S9 and S10 Figs. Interestingly, most of the genes that are key in building the antibiotic classifier, which examines the prediction strengths of individual genes, are also identified as the high/low enrichment alleles analyzed in the above genomic plots (Fig 1a, S2 Fig). Also, the distinction between antibiotics and their classes diminishes when using the binary Jaccard distance metric, which operates on a gene presence/absence basis (S7 Fig). This strengthens the conclusion that while alleles conferring multi-drug resistance are found in many cases, variation in the degree of enrichment of these MDR alleles for a particular antibiotic is a predictor of the genetic fingerprint of a particular antibiotic.

## Conclusions

We have presented a model pipeline for the analysis of gene products leading to antimicrobial resistance in *E*. *coli*. We discovered that many alleles isolated from treatment with low levels of single antibiotics conferred resistance to many antibiotics. This lends support to the hypothesis that low-dose antibiotics as used in livestock growth promotion and found in wastewater likely promote resistance to a wide range of antimicrobial compounds including last-resort therapeutics [17]. The rise of antimicrobial resistance is also important in microbial ecology, including soil [67] and human gut [68] bacteria.

Chemical tolerance in microbes is often a complex phenotype conferred by a range of genetic factors that are often not intuitive or obvious. A seminal work in chemical genomics in *E*. *coli* was recently published in which a library of over 4,000 strains including the Keio deletion library was screened under many different chemical and physical conditions [3]. In that work, individual strains were plated robotically in 1,536-well format, and colony size was investigated to determine fitness. A similar work examined the effect of a library of 4,000 *E*. *coli* genes overexpressed on plasmids challenged by a variety of chemicals [25]. While the library was assayed in multiplex in microtiter plates, characterization of alleles (by the nature of Sanger sequencing) was limited to less than 10 colonies per condition. A similar study of all Keio collection knockouts on 14 different antibiotics also required robotics and was focused on antibiotic sensitivity [26]. This study discovered multiple-resistance knockouts in the Keio collection. While there was little overlap with our data, of the 14 antibiotics used, only two were used in this study, highlighting the complexity of multi-drug resistance. A recent study focused on aminoglycoside antibiotics used adaptive evolution over hundreds of generations to examine beneficial mutations and characterized by whole genome sequencing of 240 parallel-evolved lines [6]. Several of the above-mentioned studies concluded that mutations that affected efflux pumps such as AcrAB contributed to multiple-drug resistance. Our observation of the *marR* allele observed agrees with this result, but as the scope of our search was much broader we were also able to determine multi-drug resistant alleles with mechanisms which do not necessarily have to do with efflux pump regulation as well as alleles with unknown function without whole-genome sequencing. While we did not see significant enrichment of *acrAB_up*, it is possible that overexpression of these membrane proteins is more detrimental to the cell than the efflux of the antibiotic, resulting in an overall slower growth rate.

The original application of the TRMR library used DNA microarrays and exploratory, not multivariate, analyses to characterize the genome-level responses to various conditions. However, this application required custom-made arrays corresponding to the barcodes. In addition, as demonstrated by the application of Bar-seq to a yeast deletion library [14], sequencing has many advantages over microarrays for rapid analysis of barcoded libraries, including but not limited to cost, the ability to pursue many biological replicates under various conditions in one sequencing lane, reduced crosstalk, and increased resolution on the low and high ends of detection [69]. Previously, DNA sequencing data from barcoded libraries was analyzed using packages with specialized analyses, and for smaller, number dense datasets [70]. In contrast, sparse datasets (containing many zeroes) like the one presented in this work make metagenomic techniques like the analyses in QIIME more appropriate [35]. QIIME also contains many analysis types in one package, streamlining analyses, and can easily analyze dataset sizes from small to massive [71]. As input, QIIME only requires sample metadata (e.g., antibiotic type) and a large count matrix derived from high-throughput sequencing, in which samples are the columns, and the counts of each feature (gene, microbe, etc.) are the rows.

Overall, our approach allows such analyses in multiplex at the level of growth selections (over roughly 24 to 48 hours) and now in the sequencing steps as well, allowing considerably faster, deeper, and larger laboratory population genomic dynamics studies in bacteria. Barcoding maximizes the usefulness of short reads and allows for the use of HiSeq technology to generate millions of times more data points than Sanger sequencing would allow. In addition, the barcoded and pre-defined nature of the library circumvents the need for long adaptation cycles (10–100 times fewer generations required) and whole genome sequencing. Thus, the combination of a method to map the specific effect of genes to selectable traits (TRMR), high-throughput sequencing, and streamlined bioinformatics analysis software (QIIME) provides a powerful toolbox for exploring the genetic basis of a broad variety of complex phenotypes [32]. Finally, the same methodologies of selection, high-throughput sequencing, and bioinformatic analysis are broadly applicable to experiments on chemical tolerance for any inhibitory chemical, from antibiotics to toxic metabolites to next-generation biofuels.

## Supporting Information

S1 Dataset Raw counts and enrichment scores. (XLSX)

S1 Fig Schematic of the inserted cassette in TRMR library mutants. (JPG)

S2 Fig Multi-drug sensitivity genes selected for across all eight antibiotics. TRMR “up” (blue) or “down” (red) alleles conveying the lowest enrichment for particular antibiotics are plotted relative to their location in the E. coli genome (in Mb). Alleles diminished in many or all selections are highlighted. The outer ring represents a linear combination of all eight antibiotic trials.
(JPG)

S3 Fig Percent of selected populations comprising multi-drug resistant genes. (JPG)

S4 Fig OD_600_ of liquid cultures of individual TRMR clones at various antibiotic levels. Heat map representing average optical density of triplicate cultures in MOPS-glucose media. Eight antibiotic concentrations were used at two-fold serial dilutions. Maximum concentrations in μg/mL: gentamicin:1.52, kanamycin: 30, tetracycline: 18, doxycycline: 18, carbenicillin: 177.6, ticarcillin: 124.8, cefixime: 6.5, ceftazidime: 13. Overlaid in white: Enrichment scores for each clone on each antibiotic.
(TIF)

S5 Fig Network analysis of antibiotic selections. (A) Nodes are the antibiotic type, while the black dots are the genes. If a gene is shared between the antibiotics, it pulls those nodes closer at an amount weighted by the gene’s abundance. If a gene is not shared between the antibiotics, it pulls the antibiotic sample node it is attached to towards the outside of the diagram, separating the nodes. The close clustering of the antibiotic nodes indicates many shared genes. (B) The separate clustering of the TRMR ‘up’ vs. the TRMR ‘down’ antibiotic selections indicates that very different up/down genes are selected for.
(JPG)

S6 Fig Clustering by antibiotic class is consistent regardless of normalization technique. 24 hour time point (left) and late exponential phase (OD) selections (right). The rows are the normalization methods used, which are rarefying or cumulative-sum scaling (CSS) (Paulson et al., Nature Methods, 2013).
(JPG)

S7 Fig ANOSIM R-values are consistent regardless of distance metrics. 24 hour (left) and late exponential phase (OD) selections (right). Each row represents clustering with a different distance metric. The much smaller ANOSIM R-value for the binary Jaccard selections supports the hypothesis of S3 Fig: that differences in allelic population abundances, rather than the alleles themselves, are the main variable driving the antibiotic separation.
(JPG)

S8 Fig A detailed time course selection, with many more samples, on gentamicin and kanamycin results in near perfect supervised learning classification. (A) Schematic of the types of time-course experimental setups. All of these selections were done in triplicate to control for experimental variations. (B) The majority of change to the allele population occurs in the first selection, regardless of selection type. This is shown by the large Bray-Curtis distance between Time_0 (TP0) and the selection types. Also, the second and third constant transfers (CT2, CT3) or serial dilutions (SD2, SD3) do not have much higher bars than CT1or ST1. (C) Supervised learning confusion matrix for the detailed Gentamicin and Kanamycin time course study shows no error (off diagonal classification) between the two antibiotics.
(JPG)

S9 Fig Heatmap of the log10 counts of the top 25 genes that distinguish antibiotic categories in the supervised learning classifier. Label format is Antibiotic/ Method_replicate. For example, Carb24_1 means Carbenicillin was used, it is the 24- hour selection, and it is replicate 1 of 3.
(TIF)

S10 Fig Heatmap of the log10 counts of the COG categories used in the supervised learning classifier. Labeling as in S9 Fig. COG category symbol and meaning: C—Energy production and conversion, D—Cell cycle control and mitosis, E—Amino Acid metabolism and transport, F—Nucleotide metabolism and transport, G—Carbohydrate metabolism and transport, H—Coenzyme metabolism, I—Lipid metabolism, J—Translation, K—Transcription, L—Replication and repair, M—Cell wall/membrane/envelope biogenesis, N—Cell motility, O—Post-translational modification, protein turnover, chaperone functions, P—Inorganic ion transport and metabolism, Q—Secondary structure, T—Signal transduction, U—Intracellular trafficking and secretion, Y—Nuclear structure, Z—Cytoskeleton, R—General function prediction only, S—Function unknown.
(TIF)

S1 Table Antibiotics and concentrations used in this study. (XLSX)

S2 Table Top 10 highly enriched alleles in OD selections for each antibiotic. (XLSX)

S3 Table Top 10 diminished alleles in OD selections for each antibiotic. (XLSX)

S4 Table Nonparametric ANOSIM values for important categories in this study. The R statistic represents the how different the tested categories are, with a value near zero indicating no significant difference between the groups, and a value near 1 indicating difference.
(XLSX)

S5 Table A1 top genes used for classification between the eight antibiotics. (XLS)
