# Supplementary figures and images for: Parallel Mapping of Antibiotic Resistance Alleles in Escherichia coli
Source: PLoS One. 2016 Jan 15;11(1):e0146916. doi: 10.1371/journal.pone.0146916 (PMC4714920; doi:10.1371/journal.pone.0146916)

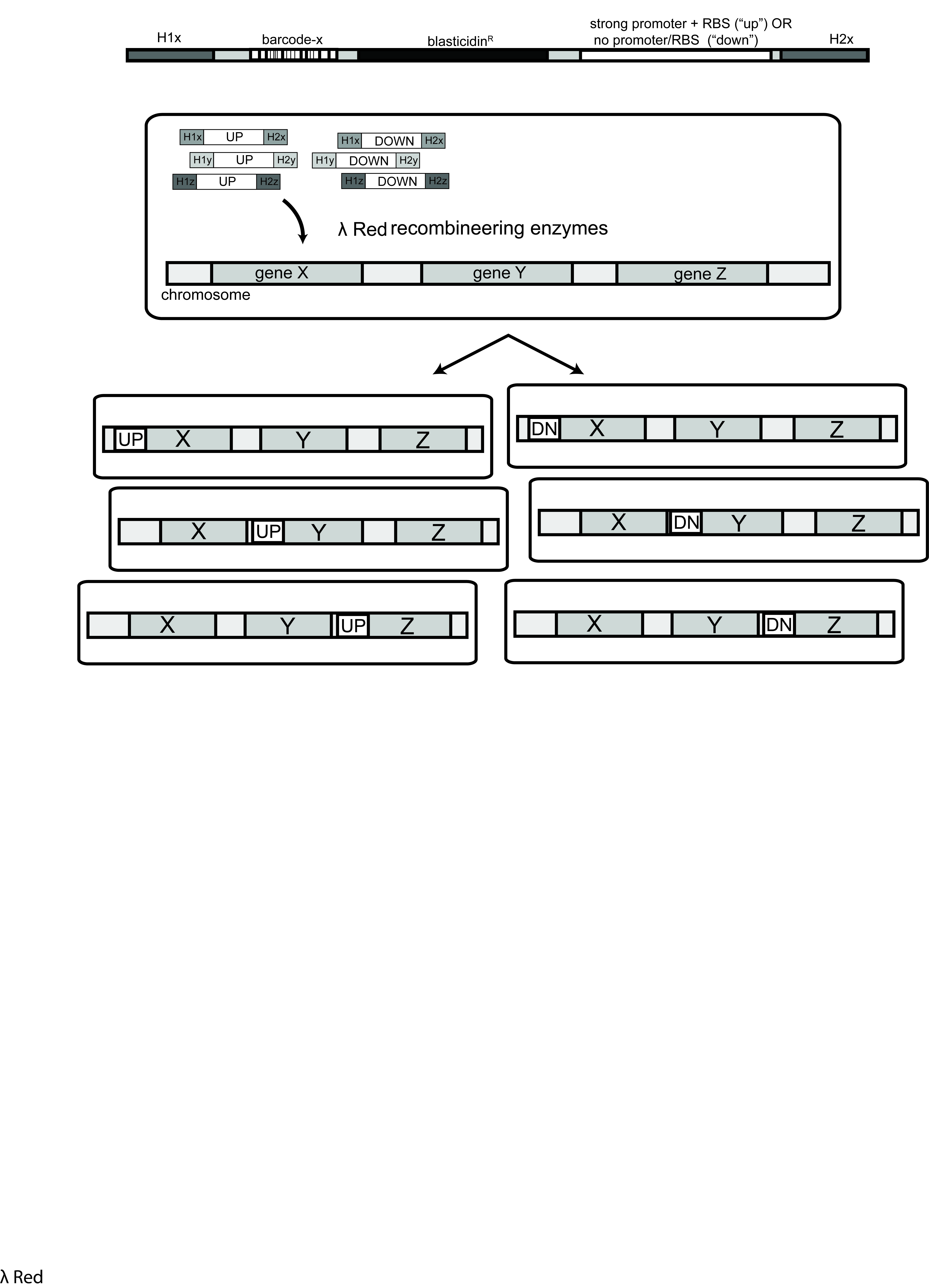

Supplement: S1 Fig — (JPG) [file pone.0146916.s002.jpg]

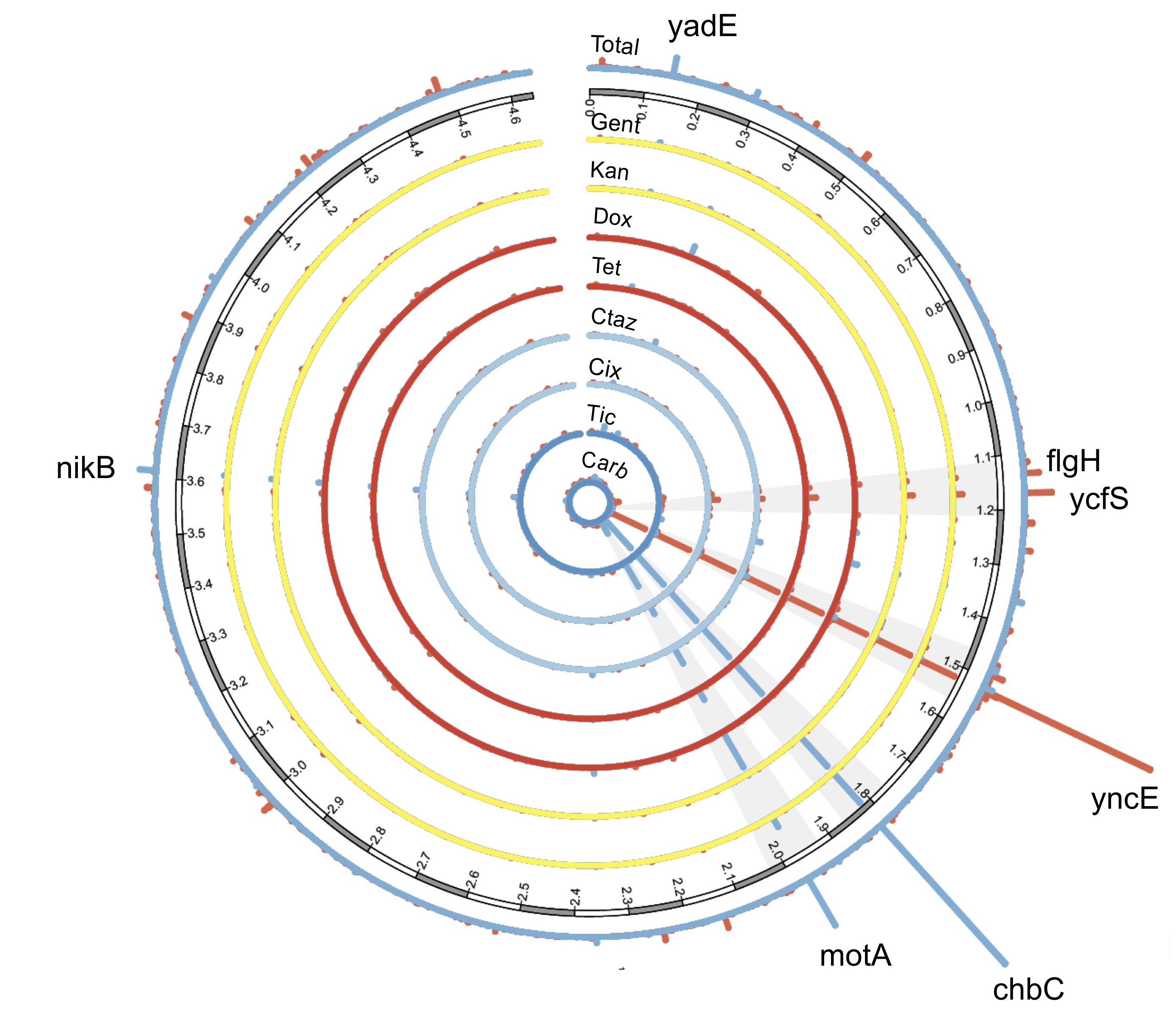

Supplement: S2 Fig — TRMR “up” (blue) or “down” (red) alleles conveying the lowest enrichment for particular antibiotics are plotted relative to their location in the E. coli genome (in Mb). Alleles diminished in many or all selections are highlighted. The outer ring represents a linear combination of all eight antibiotic trials. (JPG) [file pone.0146916.s003.jpg]

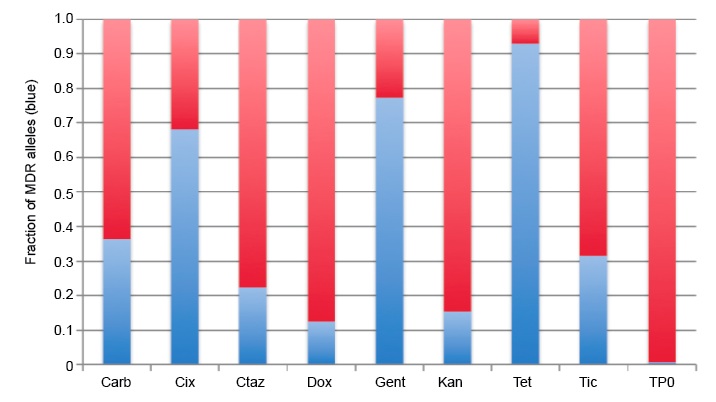

Supplement: S3 Fig — (JPG) [file pone.0146916.s004.jpg]

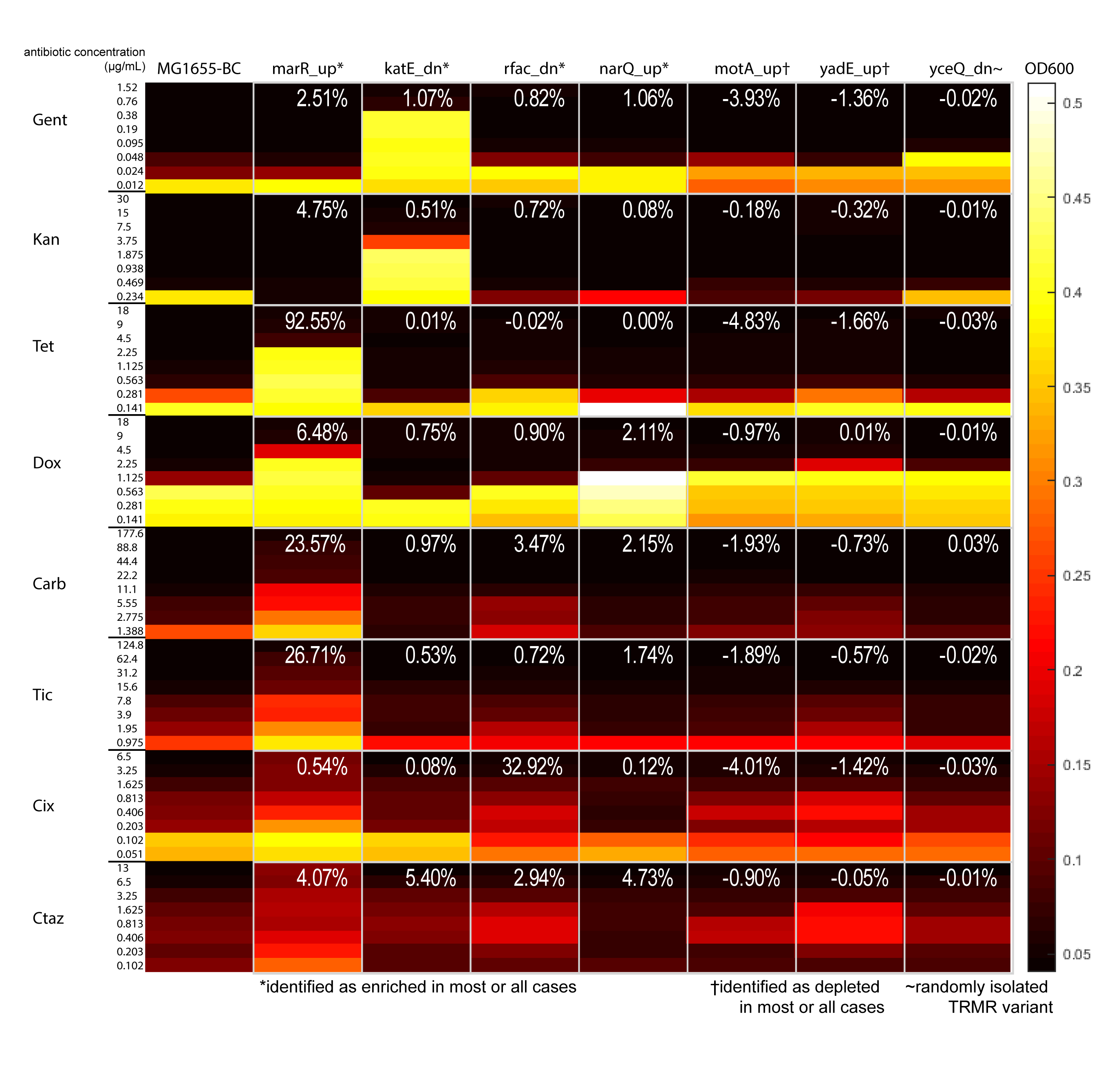

Supplement: S4 Fig — Heat map representing average optical density of triplicate cultures in MOPS-glucose media. Eight antibiotic concentrations were used at two-fold serial dilutions. Maximum concentrations in μg/mL: gentamicin:1.52, kanamycin: 30, tetracycline: 18, doxycycline: 18, carbenicillin: 177.6, ticarcillin: 124.8, cefixime: 6.5, ceftazidime: 13. Overlaid in white: Enrichment scores for each clone on each antibiotic. (TIF) [file pone.0146916.s005.tif]

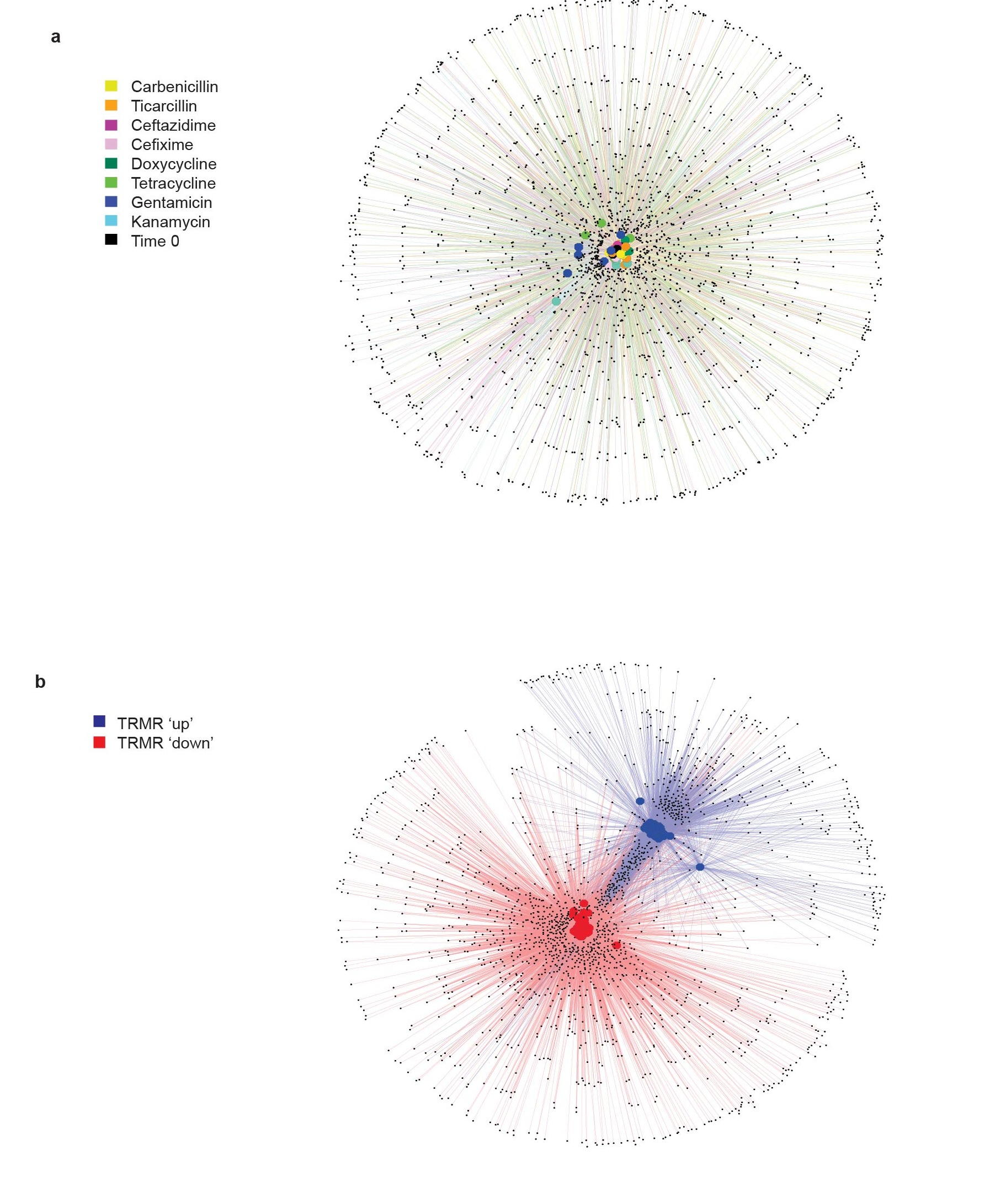

Supplement: S5 Fig — (A) Nodes are the antibiotic type, while the black dots are the genes. If a gene is shared between the antibiotics, it pulls those nodes closer at an amount weighted by the gene’s abundance. If a gene is not shared between the antibiotics, it pulls the antibiotic sample node it is attached to towards the outside of the diagram, separating the nodes. The close clustering of the antibiotic nodes indicates many shared genes. (B) The separate clustering of the TRMR ‘up’ vs. the TRMR ‘down’ antibiotic selections indicates that very different up/down genes are selected for. (JPG) [file pone.0146916.s006.jpg]

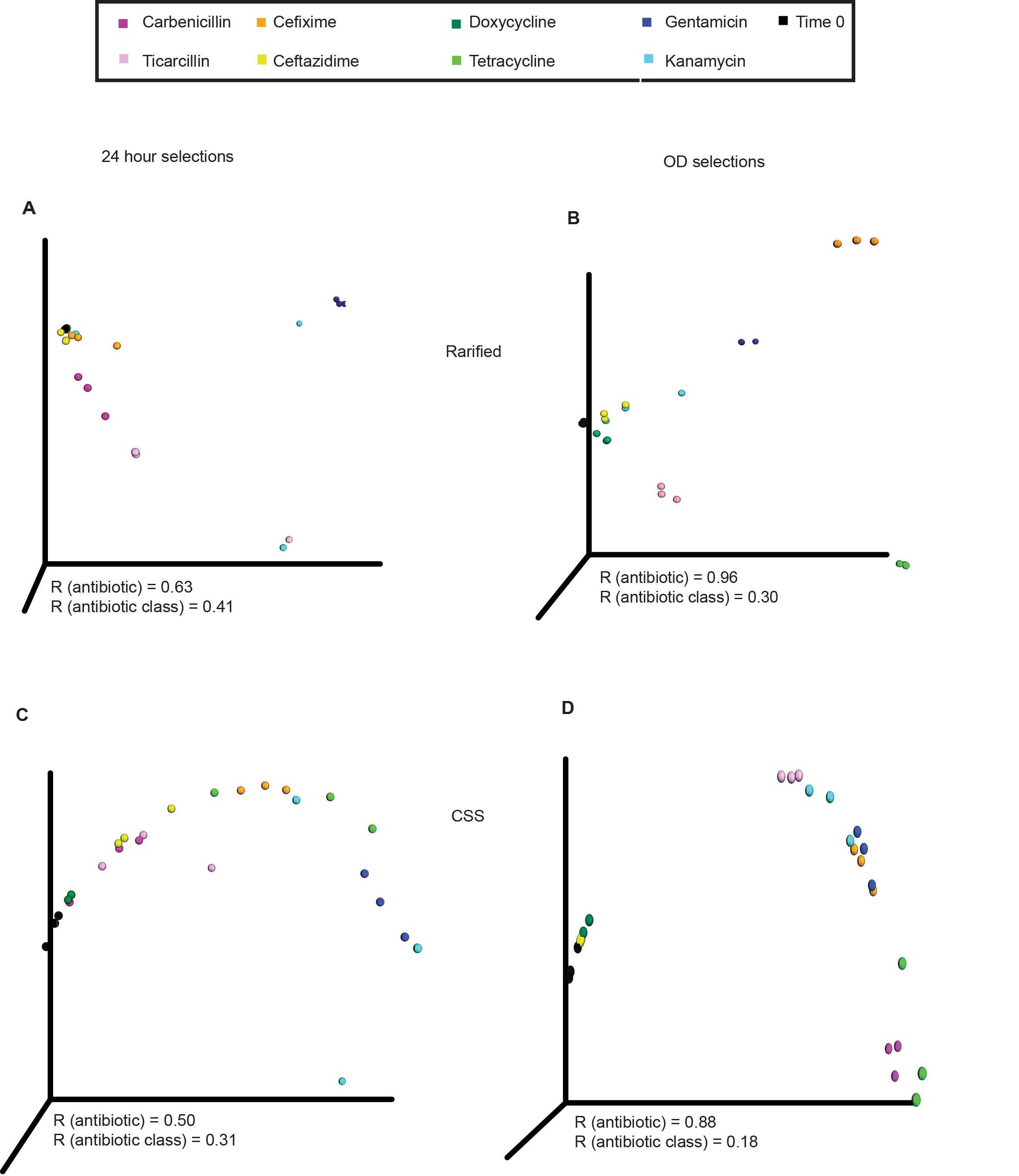

Supplement: S6 Fig — 24 hour time point (left) and late exponential phase (OD) selections (right). The rows are the normalization methods used, which are rarefying or cumulative-sum scaling (CSS) (Paulson et al., Nature Methods, 2013). (JPG) [file pone.0146916.s007.jpg]

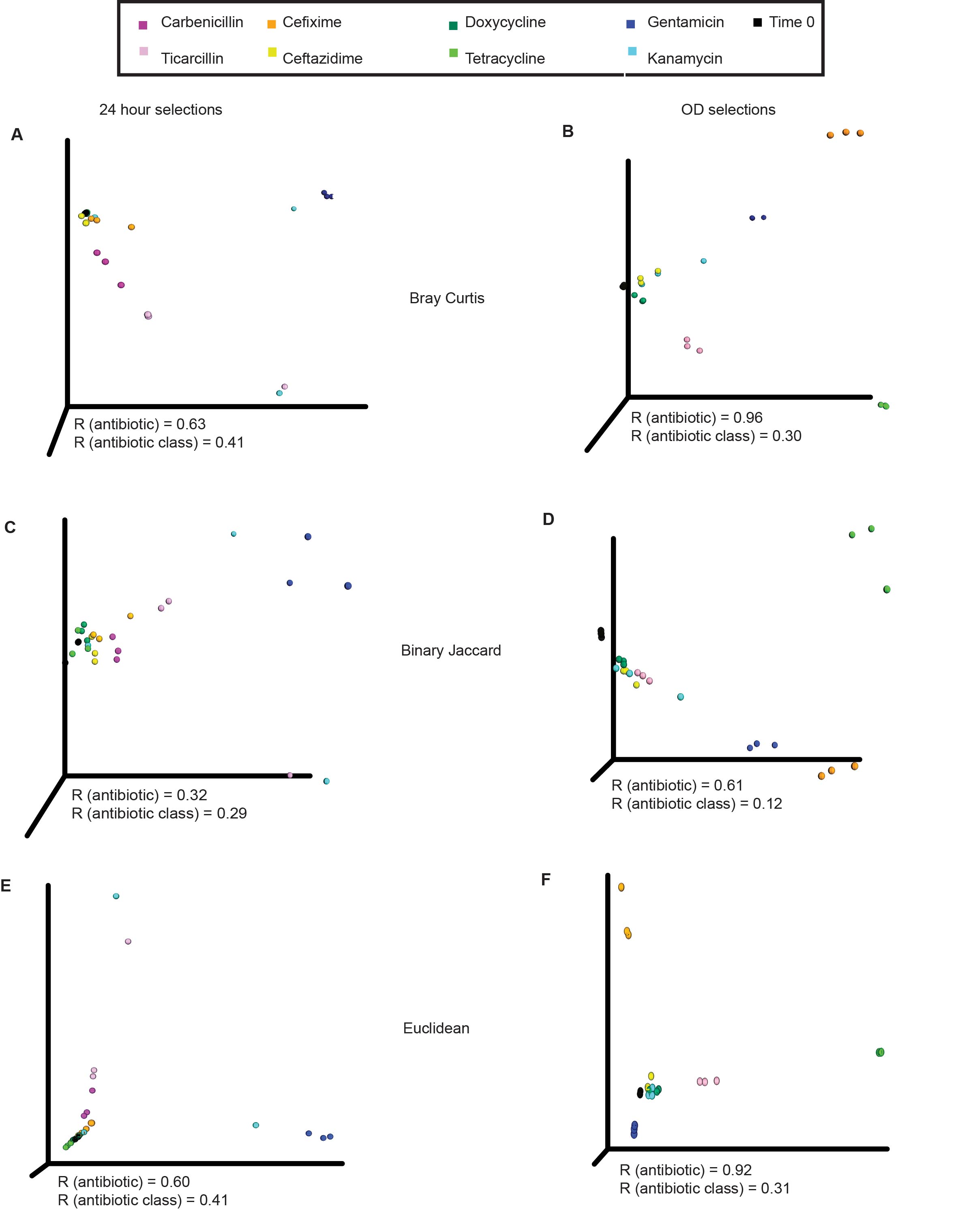

Supplement: S7 Fig — 24 hour (left) and late exponential phase (OD) selections (right). Each row represents clustering with a different distance metric. The much smaller ANOSIM R-value for the binary Jaccard selections supports the hypothesis of S3 Fig: that differences in allelic population abundances, rather than the alleles themselves, are the main variable driving the antibiotic separation. (JPG) [file pone.0146916.s008.jpg]

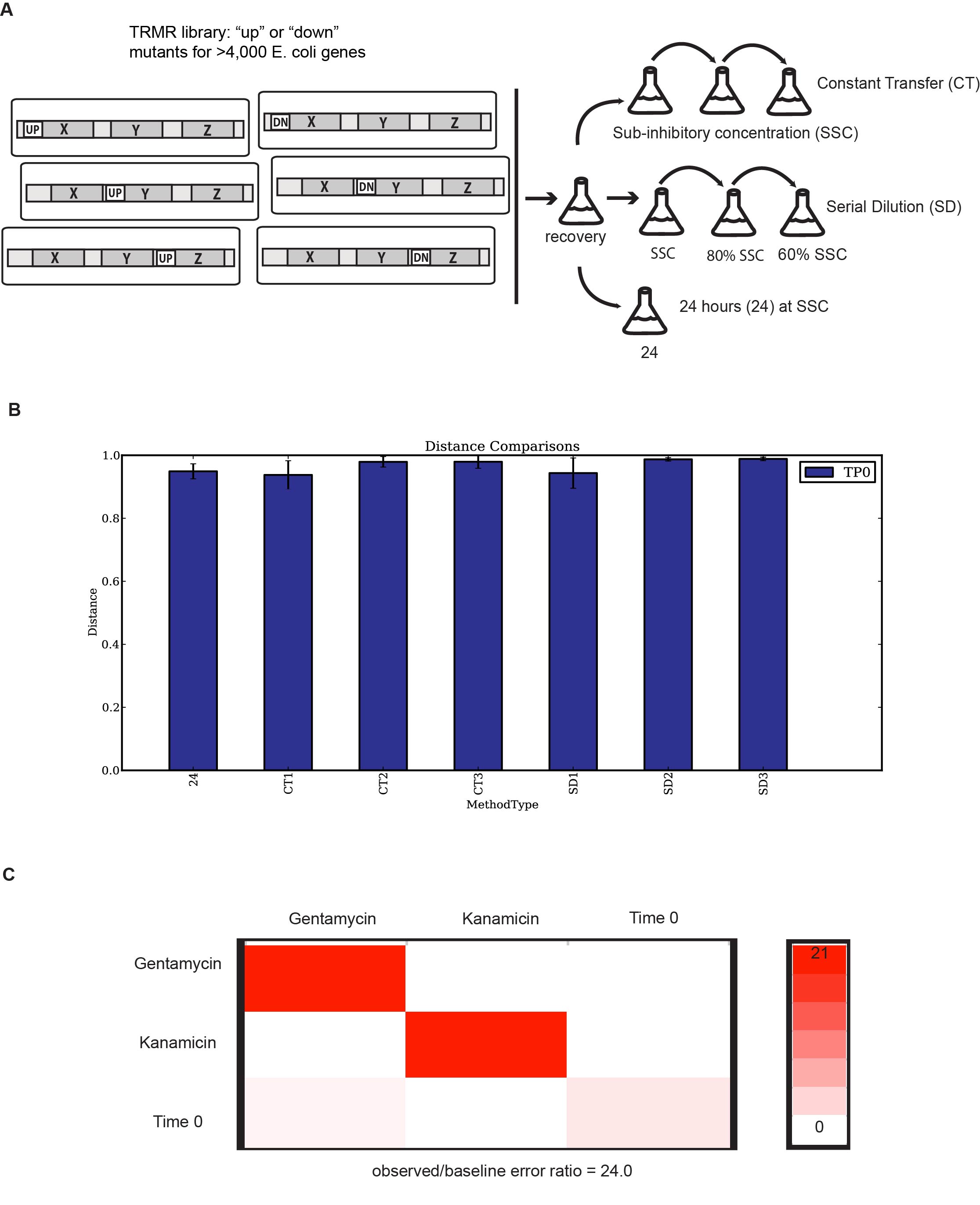

Supplement: S8 Fig — (A) Schematic of the types of time-course experimental setups. All of these selections were done in triplicate to control for experimental variations. (B) The majority of change to the allele population occurs in the first selection, regardless of selection type. This is shown by the large Bray-Curtis distance between Time_0 (TP0) and the selection types. Also, the second and third constant transfers (CT2, CT3) or serial dilutions (SD2, SD3) do not have much higher bars than CT1or ST1. (C) Supervised learning confusion matrix for the detailed Gentamicin and Kanamycin time course study shows no error (off diagonal classification) between the two antibiotics. (JPG) [file pone.0146916.s009.jpg]

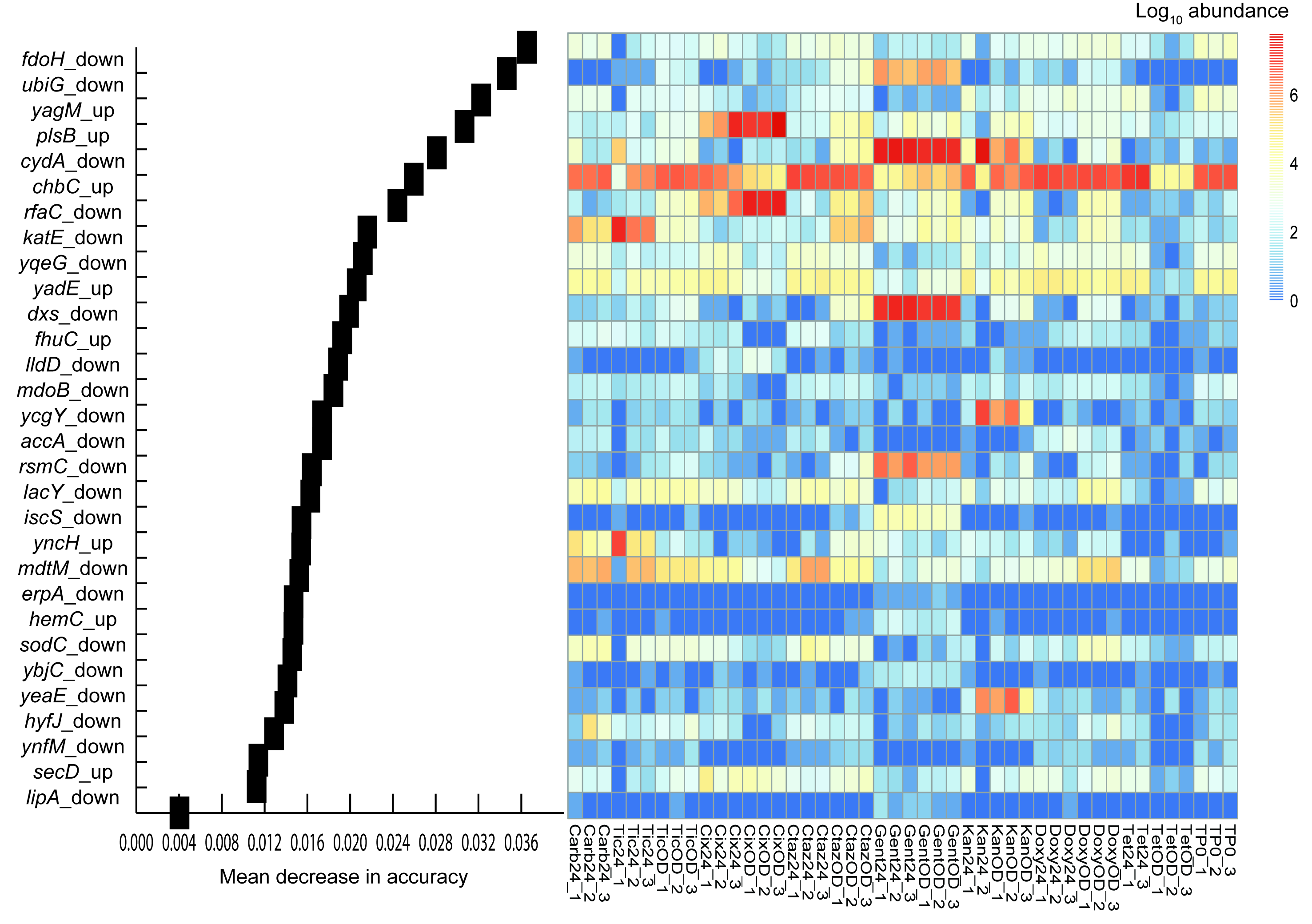

Supplement: S9 Fig — Label format is Antibiotic/ Method_replicate. For example, Carb24_1 means Carbenicillin was used, it is the 24- hour selection, and it is replicate 1 of 3. (TIF) [file pone.0146916.s010.tif]

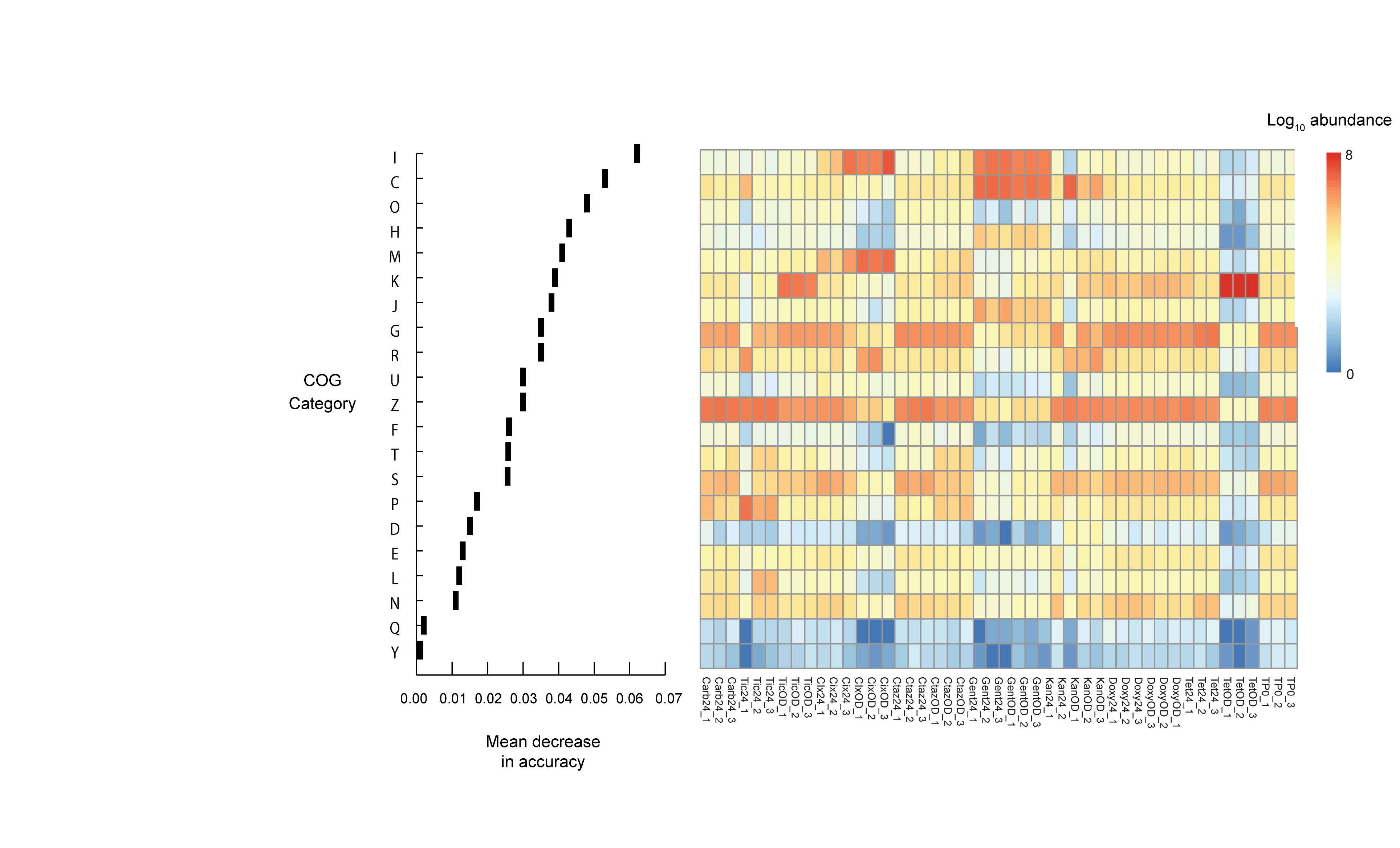

Supplement: S10 Fig — Labeling as in S9 Fig. COG category symbol and meaning: C—Energy production and conversion, D—Cell cycle control and mitosis, E—Amino Acid metabolism and transport, F—Nucleotide metabolism and transport, G—Carbohydrate metabolism and transport, H—Coenzyme metabolism, I—Lipid metabolism, J—Translation, K—Transcription, L—Replication and repair, M—Cell wall/membrane/envelope biogenesis, N—Cell motility, O—Post-translational modification, protein turnover, chaperone functions, P—Inorganic ion transport and metabolism, Q—Secondary structure, T—Signal transduction, U—Intracellular trafficking and secretion, Y—Nuclear structure, Z—Cytoskeleton, R—General function prediction only, S—Function unknown. (TIF) [file pone.0146916.s011.tif]
